# Children with Disabilities in Canada during the COVID-19 Pandemic: An Analysis of COVID-19 Policies through a Disability Rights Lens

**DOI:** 10.3390/children10060942

**Published:** 2023-05-26

**Authors:** Keiko Shikako, Raphael Lencucha, Matthew Hunt, Sébastien Jodoin-Pilon, Ananya Chandra, Anna Katalifos, Miriam Gonzalez, Sakiko Yamaguchi, Roberta Cardoso, Mayada Elsabbagh, Anne Hudon, Rachel Martens, Derrick Cogburn, Ash Seth, Genevieve Currie, Christiane Roth, Brittany Finlay, Jennifer Zwicker

**Affiliations:** 1 School of Physical and Occupational Therapy, McGill University, 3654 Prom Sir-William-Osler, Montréal (D4), Quebec, QC H3G 1Y5, Canada; 2 Faculty of Law, McGill University, Quebec, QC H3A 1W9, Canada; 3 Department of Neurology and Neurosurgery, McGill University, Quebec , QC H3A 1A1, Canada; 4 Health Center Research Institute, McGill University, Quebec, QC H4A 3J1, Canada; 5 Montreal Neurological Institute, McGill University, Quebec, QC H3A 2B4, Canada; 6 School of Rehabilitation, University of Montreal, Montreal, QC H3N 1X7, Canada; 7 Kids Brain Health Network, Burnaby, BC V5A 4W9, Canada; 8 School of International Service, Institute on Disability and Public Policy, American University, Washington, DC 20016, USA; 9 School of Public Policy, University of Calgary, Calgary, AB T2P 1H9, Canada

**Keywords:** children with disabilities, disability rights, human rights, mental health, COVID-19

## Abstract

Children with disabilities were especially vulnerable during the COVID-19 pandemic, and policies designed to mitigate its effects were limited in addressing their needs. We analyzed Canadian policies related to children with disabilities and their families during the COVID-19 pandemic to identify the extent to which these policies aligned with the United Nations Convention on the Rights of Persons with Disabilities (UN CRPD) and responded to their mental health needs by conducting a systematic collection of Canadian provincial/territorial policies produced during the pandemic, building a categorization dictionary based on the UN CRPD, using text mining, and thematic analysis to identify policies’ alignment with the UN CRPD and mental health supports. Mental health was addressed as a factor of importance in many policy documents, but specific interventions to promote or treat mental health were scarce. Most public health policies and recommendations are related to educational settings, demonstrating how public health for children with disabilities relies on education and community that may be out of the healthcare system and unavailable during extended periods of the pandemic. Policies often acknowledged the challenges faced by children with disabilities and their families but offered few mitigation strategies with limited considerations for human rights protection.

## 1. Introduction

Individuals with disabilities have encountered unique challenges during the COVID-19 pandemic. In addition to increased medical risks associated with COVID-19, persons with disabilities have faced challenges accessing health information, lockdown measures that failed to consider their specific needs, and disruption of essential services and supports [1,2]. Many encountered additional social and economic barriers during the pandemic, such as heightened food insecurity, higher rates of unemployment, and worsened mental health [3].

Many policy responses to COVID-19 failed to consider or provide targeted support for different vulnerable populations, especially those who experience multiple layers of intersectional marginalization, such as children with disabilities and their families [4,5]. Other issues arising from intersectional marginalization have also become more apparent during the pandemic: Persons with disabilities have higher rates of institutionalization, social isolation, loss of essential services, and denial of access to healthcare as a result of either government responses during the pandemic or systemic inequities that existed before the pandemic [6]. Children and youth with disabilities are frequent users of residential services that are meant for the elderly [7,8] and, as such, were exposed to a higher risk of contamination, isolation, and denied contact with their families [9,10]. Evidence suggests, for instance, that lockdown guidelines for long-term care facilities were linked with exacerbated aggression in residents with intellectual disabilities compared to those without [11]. A lack of targeted policies and public health measures resulted in an uneven distribution of essential services and deleterious consequences for their health and well-being.

An analysis of the disability response to COVID-19 in 14 countries and its alignment with the United Nations Convention on the Rights of Persons with Disabilities (UN CRPD) found that while government responses acknowledged disability challenges, there was a scarcity of concrete action plans to alleviate any of these concerns. These national-level documents rarely went beyond stating a need for action and awareness. In many settings, the vaccine rollout also failed to appropriately prioritize persons with disabilities [12].

Children with disabilities face disproportionate challenges and require special protections before, during, and after the pandemic [13]. When compared to adults with disabilities or same-age peers without disabilities, children with disabilities are at a higher risk for socioeconomic hardship and homelessness, poor nutrition, domestic violence, sexual exploitation, higher stress and anxiety, and cyberbullying [14,15]. Children with disabilities are specifically recognized as facing harsher consequences of emergencies and natural disasters, especially when education and routines are compromised [16].

Many of the challenges facing persons with disabilities during the pandemic are associated with and contribute to mental health challenges. Disruptions to daily routines, including necessary care and support, coupled with socially isolating public health measures, contribute to the risk of mental health challenges. Several international organizations, including the World Health Organization (WHO) and United Nations International Children’s Emergency Fund (UNICEF), and international disability civil society organizations released disability-inclusive policy recommendations during the pandemic [17,18,19,20,21,22] that emphasized a rights-based approach in which response policies are informed by the provisions of the United Nations Convention on the Rights of Persons with Disabilities (UN CRPD). Created by the United Nations in 2006, the UN CRPD requires all member states to protect and promote the human rights of persons with disabilities in a variety of enumerated areas, including health, education, and legal and social protections. The COVID-19 recommendations included priority testing for persons with disabilities, pathways to equitable access to healthcare, guidelines pertaining to personal protective equipment, and procedures to ensure access to relevant information. The recommendations also addressed considerations beyond disease prevention and health services, such as guidance on how to alleviate and address social isolation, ensure inclusive and accessible education for students (including concerns, such as wireless internet access and appropriate adaptive technology in the context of distanced learning), and minimize socioeconomic inequalities.

The WHO recognizes that the pandemic negatively impacted mental health at a populational level and presented heightened risks to those already living with mental illness [23]. Furthermore, ref. [24] identified women, children, and adolescents as facing particularly heightened mental health challenges during the pandemic. The recommendations made to mitigate these challenges were for countries to institute widespread, accessible, and equitable mental health resources for all and to endorse mental health as a needed key component of recovery efforts. In particular, for children with disabilities, UNICEF has drawn attention to their vulnerability and urged for data collection and research to be conducted in order to better understand the experiences of children with disabilities during the pandemic, including the inadvertent effects of lockdowns and service interruptions on the exacerbation of anxiety and behaviour problems [25].

This study explores the alignment of COVID-19 policy responses in Canadian provinces and territories with the UN CRPD indicators, with particular emphasis on how policy responses addressed the mental health of children with disabilities.

## 2. Materials and Methods

We conducted an environmental scan of policies generated by provincial and territorial governments during the COVID-19 pandemic and used text mining methodology and thematic analysis to assess this content in relation to the provisions of the UN CRPD and the supports for the mental health of children with disabilities. This participatory project included an advisory group composed of youth with disabilities, parents of children with disabilities, and representatives of organizations of persons with disabilities, who advised on all steps of the research project.

### 2.1. Procedures

#### 2.1.1. Data Collection

We searched provincial and territorial government websites, including the websites of individual ministries (e.g., Health, Families, Children), to identify all COVID-19 pandemic-related policies (see Appendix A for a list of government websites searched). We identified search keywords in consultation with the advisory group for the project. The initial keywords included: COVID-19, child/children, disabilities, mental health, and families. The inclusion criteria were policies that: (1) pertained to the pandemic or lockdown situation and were enacted between September 2020 and April 2021; (2) made specific reference to persons with disabilities (not exclusively children) or their family members/caretakers (specific categories of disabilities, such as “intellectual disabilities” or “autism”, met this criterion; synonyms for disabilities, such as “handicapped” or “persons/students with special needs”, met this criterion; broader categories, such as “vulnerable populations” or “marginalized communities”, that include other people in addition to persons with disabilities did not meet this criterion); and (3) inclusive of youth who are 24 years of age or younger (policies were only excluded on the basis of this criterion if they specified an age range that did not overlap with 0–24 years of age, such as policies targeting geriatric/older persons with disabilities). We modified our approach for documents from the three territories. Policies from the territories were included if they were published online by territorial governments or ministries in either English or French, and were still in effect during the COVID-19 pandemic (i.e., policies that had not been suspended or deemed irrelevant due to the pandemic or lockdown measures) and made specific reference to persons with disabilities (this criterion was applied the same way as it was in the provinces). Once documents were identified, they were downloaded, or the entire webpage was saved as a document for data extraction. Each document was then screened by two reviewers using the inclusion criteria. Disagreements in inclusion were discussed with the research team. Due to the low volume of data available and the necessity for flexibility in the territories’ context (policies in the territories reflect the service structure that is more centralized and less disability-specific; e.g., children with disabilities are often included in regular classrooms, pediatric and adult services may be combined due to the lower number of children with disabilities, and possible COVID-19 cases, which has implications in the types of public health restrictions and specificity of policies), and the age parameters were suspended in the territories. The parameters for date and relevance to the coronavirus pandemic were also broadened to include documents related to children with disabilities produced prior to the pandemic that would still be valid during the pandemic (e.g., mental health services and supports) exclusively in the territories, considering the population limitations (low N) and the number of documents initially identified.

#### 2.1.2. Data Extraction

The team developed a screening form to select documents to be retained for analysis (Appendix B) and a data extraction sheet to collect information from the retained articles for descriptive analysis (Table 1).

### 2.2. Analysis

#### 2.2.1. Categorization Models

The policies were first analyzed using the Cross-Industry Standard Process for Data Mining (CRISP-DM) approach for text mining. The CRISP-DM comprises six stages: problem formulation, data collection, data preparation, model development, analysis, and deployment [26]. We used the WordStat tool (Provalis Research, Montreal, Canada) created by Provalis Research, which relies on a “bag of words” technique, wherein text is represented as a multiset of its words, and grammar or order of words is largely disregarded. WordStat offers a feature that allows users to write categorization dictionaries that can search the text for specific patterns of words and identify how frequently each category appears in a given data set.

We used WordStat to write two dictionaries. The first categorization model was developed specifically on mental health objectives. It was developed by the research team, which included researchers in mental health, child health, and health and social policy, and in collaboration with the advisory group. The team operationalized mental health according to the WHO definition and in relation to the COVID-19 pandemic, which was based on symptoms (anxiety, depression, stress, behaviour problems, and sleep problems) and service provisions (mental health supports and services) [27]. The mental health dictionary was categorized according to the three topics in this dictionary: Stressors, Barriers, and Symptoms/Outcomes (see Appendix C for visualization of the Mental Health categorization model). The Barriers category was subdivided into four types of barriers: Attitudinal, Environmental, Medical, and Structural. Each of these categories and subcategories was then populated with proximity rules that defined these concepts or iterated examples of concepts. The proximity rules function by using anywhere between two to five words or phrases that are coded such that if they are found in a particular pattern or in proximity to one another, then the program would save that sentence as a reference to the category or subcategory that the rule was written under.

**Figure 1:**
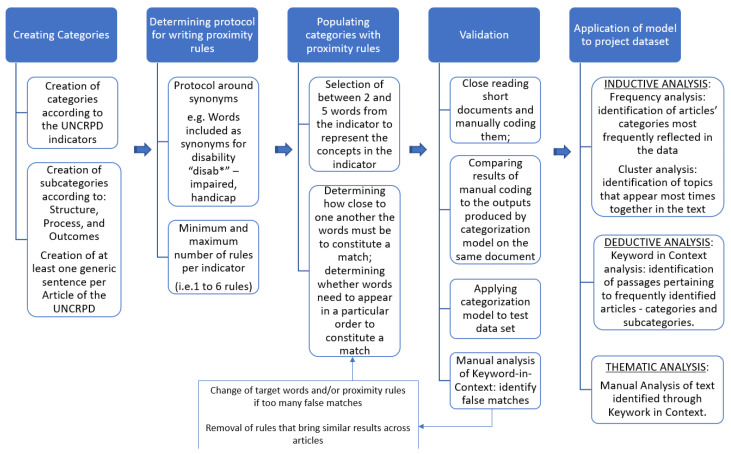
Summary description of categorization model development.

The second dictionary is a categorization model based on the UN CRPD Bridging the Gap indicators proposed by the Office of the United Nations High Commissioner on Human Rights (OHCHR) [28]. These indicators address the detailed structures, processes, and outcomes necessary to implement and monitor the implementation of each UN CRPD article (see Appendix D for visualization of the UN CRPD categorization model). This categorization dictionary was developed by the team [29] and has been used and refined in other projects [30]. We assigned each article of the first 33 UN CRPD articles, for which the Bridge the Gap indicators have been developed (some of which were grouped together: articles 1, 2, 3, and 4 shared one set of indicators, as did articles 15 and 17) between 15 and 45 indicators of implementation, depending on the number of indicators designated by the OHCHR. Each article of the UN CRPD has between two and four topical subcategories in the OHCHR indicators framework (e.g., under Article 11: Situations of Risk and Humanitarian Emergencies subcategories are: “prevention and preparedness”, “rescue and response”, and “recovery, reconstruction, reconciliation”). We attributed a separate category under the UN CRPD indicators dictionary to each article, then subcategorized it into “Structure”, “Process”, and “Outcome”, according to the OHCHR framework. Next, we created a subcategory for the subtopics that were specific to that article’s indicators. Finally, each indicator was given anywhere between one and six proximity rules—which allowed us to search the data for phrases, sentences, paragraphs, or pages that reference the content of that indicator. Each article and subcategory had a different number of rules based on the number of indicators in that category and their complexity. Please refer to Figure 1 for an illustration of how the categorization model was created, and refer to [29] for a full description. We used this model to assess the extent to which indicators across the entirety of the UN CRPD were represented in the policy documents collected.

#### 2.2.2. Descriptive and Content Analysis

We ran descriptive statistics in WordStat to define the frequency of articles in the UN CRPD model, and mental health model topics were matched in the data. We selected the four UN CRPD articles that had the most content relating to them (as matched through our categorization model) for in-depth analysis. There is no methodological standard for text mining scanning followed by in-depth “manual” analysis. Our team, including information systems experts in text mining and researchers experienced in policy analysis, determined through consensus that this approach would reflect the most salient content to address our research question [29,30]. We then used the WordStat function “Key Words in Context”, in which the target keywords that define each article categorization rule are subject to a close read analysis, along with the text in which the keyword was identified. In this way, we conducted a close reading of the text surrounding the KeyWords in context, consulting the entire policy documents as needed (i.e., to clarify a concept that may not have been fully captured in the KeyWords in context extract) and conducted a thematic analysis of this content. Below, we: 1. describe the content of the policies that relate to these articles captured through the UN CRPD model, and 2. present the thematic analysis carried on the text captured through the mental health model.

## 3. Results

### 3.1. Description of Policy Documents

**Figure 2:**
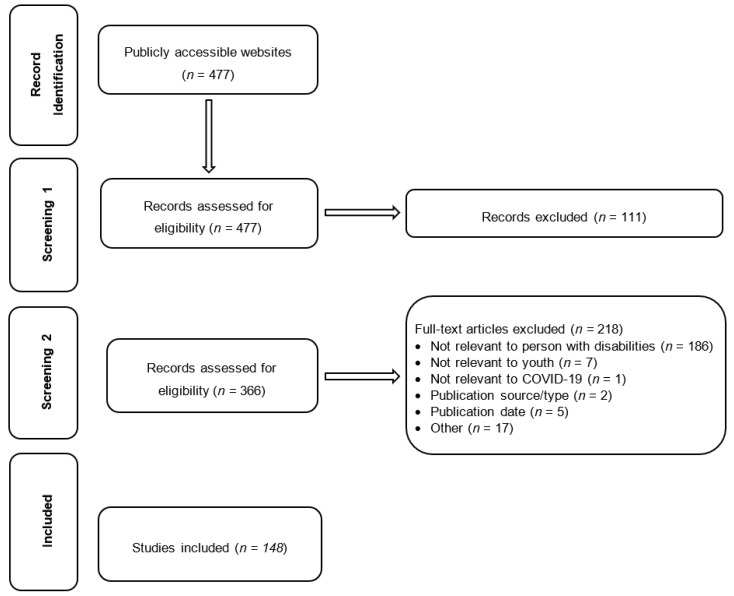
Document selection flowchart.

Figure 2 shows the review flow chart. A total of 148 policies were identified across the 10 Canadian provinces and 3 territories during the data collection period of September 2020 to April 2021. There was considerable variation in the number of documents released in each region. Quebec (*n* = 37), Ontario (*n* = 20), and Manitoba (*n* = 16) produced the highest number of policies, while the three territories, Nova Scotia, and Saskatchewan published the fewest (*n* < 5). Table 2 presents the provincial distribution of policies and the main characteristics of the documents identified.

Most documents (57/133) provided recommendations for service improvements that were not specific to mental health but could peripherally relate to or apply to mental health services. For example, Ontario (ON) issued a document outlining recommendations with the goal of limiting transmission of COVID-19 for people residing or working in congregate living settings, including mental health institutions [31]. Another example was documents that provided information about respite care, a strong contributor to families’ mental health, and emergency services for families of children and youth with special needs, which could include mental health emergency services. The documents also outlined overall policy goals, including, for example, information about the reorganization and prioritization of activities and programs associated with the Ministry of Health and Social Services in Quebec (QC). Another common area of focus involved guidance on education, such as two back-to-school plans from Nova Scotia (NS) and Nunavut (NV) for the 2020–2021 school year that detail risk mitigation strategies for schools. While these policies are not specific to mental health, the return to school was a necessary step toward the resumption of services for many children with disabilities. These services include mental health supports offered through schools, and for that reason, the related policies were included for analysis. 

### 3.2. Mental Health Analytical Model

The mental health model was used to identify the policy content specific to the conditions that contribute to mental health in children, including those with disabilities, and the possible mental health impacts of COVID-19 protection measures. The majority of the policies identified in this category relate to the education sector. Health and mental health are impacted by disruptions in the routines of daily life, including protective measures implemented in school settings or shifts to distance learning. The policies often emphasized the need to recognize and respond to the added stress that these disruptions might elicit. For example, this statement from a policy document from the province of NL reflects this emphasis on the recognition of mental health impacts of the pandemic: “*They may feel anxious or nervous and be worried about the virus*” [32]. The shift to distance learning was recognized as a factor impacting children’s immediate and long-term developmental outcomes by a small number of provinces and territories. For example, a document from the YK territorial government highlighted that distance learning “*can have negative short and long-term impacts on educational outcomes, achievement levels, and school drop-out rates*” [33]. The government of the NWT extended the recognition of the potential for this shift to distance learning to have a particularly disruptive impact on children with *‘complex needs’* and their families and recognized the need for an individualized, needs-based approach: “*Children with complex needs may have a particularly difficult time coping with this sudden change, and that also asks a lot of parents. The focus for learning will need to be different for everyone*” [34].

The negative impact of long-term isolation, tied in part to disruptions to in-person school-based routines, was another commonly identified risk factor for mental distress. A policy document produced by the government of MB noted, “*For Manitobans with disabilities, including seniors, ongoing isolation presents additional risk to their mental health, contributing to conditions such as depression and anxiety*” [35]. It was also noted in a document published by the AB government that “*Mental health has also been a concern for children during pandemics and school isolation…*” [36], recognizing that schools are venues that promote mental health, and the absence of school-based activities adds to the mental health risks. The shift to distance learning was also presented as a limitation for teachers to identify distress in students, which would be identified during in-person interactions. At the same time, other provinces and territories identified risk factors associated with in-person activities, including stress and anxiety associated with the risk of infection and stigma associated with decisions pertaining to mask-wearing. Additionally, anxiety surrounding the possible loss of a care provider for a child with a disability was noted in a document produced by the government of BC, “*Family members may experience fear of how their loved one will be cared for if they themselves become ill with COVID-19 and are no longer able to provide care*” [37]. The government of New Brunswick was the only province or territory that generated guidance specific to caregiver fatigue, highlighting the extra stress that may be placed on caregivers while adapting to COVID-19 measures [38].

### 3.3. UN CRPD Analytical Model

**Figure 3:**
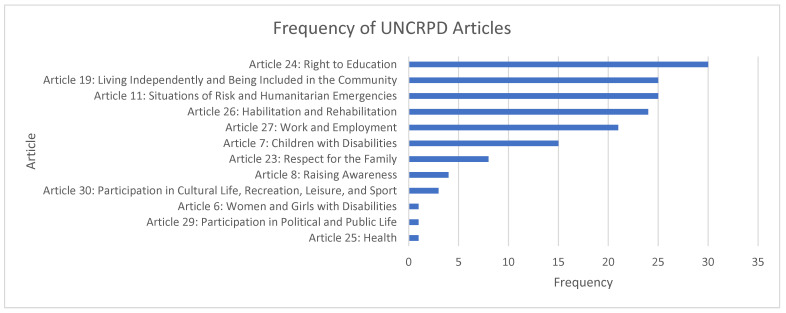
Frequency of matches with UN CRPD Indicators categorization model.

The content of the policy documents was categorized into (i.e., matched with) 11 of the 33 articles of the UN CRPD. The remaining articles were not identified by our model in any of the documents. Figure 3 illustrates the frequency with which the content was matched with the 11 articles. Article 24 (Education) was the most frequently matched article in the policy documents, followed by Article 11 (situations of risk and humanitarian emergencies), Article 19 (Living independently and being included in the community), and Article 26 (Habilitation and Rehabilitation). Articles related to work and employment (Article 27), children with disabilities (Article 7), and respect for the family (Article 23) were also matched in the documents, and raising awareness (Article 8) and participation (Article 30).

Below, we describe the policy content that was associated with each article. Here, we have chosen to report on Articles with a matched content frequency of 25 or greater (i.e., at least 25 sentences, paragraphs or pages that matched the UN CRPD article category), which included Articles 24, 11, 19, and 26. Table 3 describes these UN CRPD Articles, the summary of the thematic analysis conducted on documents captured under these Articles, and the provinces and territories that had policy documents contributing to the Article.

#### 3.3.1. Article 24: Education

The most frequently matched article was Article 24. Article 24 of the UN CRPD states that all persons with disabilities are entitled to education and lifelong learning that is cognizant of their developmental needs and human potential, that enables their full participation in society, that provides an inclusive environment within their own communities, reasonably accommodates their educational needs, and recognizes and promotes the cultural value of languages, such as sign language and braille [39]. This article’s indicators refer to structures that must be put in place to guarantee that “*persons with disabilities receive the support required, within the general education system, to facilitate their effective education*” [38], including individualized support measures for academic, physical, and social development and inclusion. There was widespread representation across the regions within this article. Documents from AB, BC, NL, NS, QC, SK, NV, and YK had concepts that were captured in the analytical category. This represents the importance of the education system in the inclusion of children with disabilities and reinforces how general services and supports for children with disabilities are/should be organized in the education sector.

Policies that were matched into this article addressed a variety of topics related to educational settings. The main themes identified in the documents captured under Article 24 included: (1a) services provided in school settings and intersectionality; (1b) considerations for the alternative learning methods and infrastructure needed to maintain education during disruptions; and (1c) safety and training of school staff to continue education provisions during the pandemic.

(1a) Services provided in school settings and intersectionality: One of the common considerations found across policies was the role that schools perform in providing daily necessities for children and families, including rehabilitation services for children with disabilities and basic food and welfare for marginalized children, including those with disabilities. Considerations for intersectionality, such as attention to the needs of children with disabilities who live in poverty, were also identified in some documents. One example was found in an AB policy document [36], which discussed possible gaps in service provision and the potentially harmful consequences for families that depended on services that could not be provided in the distance-learning environment in an equitable manner: “*Low income families may be disproportionately compromised by remote schooling due to challenges of computer and internet resources, adequate space, and worsened food insecurity with the loss of school-based programs*” [36].

Intersectionality was also considered in the education-related policies for children who are marginalized and racialized when developing plans and programs for the post-pandemic period or as some restrictions were lifted: “*The plan for the 2020–21 school year will be guided by the principles of inclusive education as outlined in Nova Scotia’s Inclusive Education Policy1, which will come into effect September 2020. There will be a focus on equity by supporting students who are historically marginalized and racialized (African Nova Scotian and Mi’kmaw students)*” [40].

Policies from QC considered how school staff could help identify the health needs of children with disabilities who did not receive school-based rehabilitation services for a prolonged period: “*The managers responsible for the establishment […] should be responsible for the actions of the establishment in relation to the measures taken to fight deconditioning of persons who have a physical disability, an intellectual disability or autism spectrum disorders*” (Translated from French) [41].

(1b) Considerations for alternative learning methods: Guidance policies highlighted the need for schools to assess the accessibility of technology tools to facilitate remote learning: “*Government and school districts will continue to work to identify options for students who have limited internet availability or other barriers to online learning*” [42]. Some documents also described resources that were put in place to support students’ learning amidst the disruptions of the pandemic. Supports offered were not exclusive for children with disabilities, and little information was available in the policy documents about specific accommodations or additional supports addressing the needs of children with disabilities in the use of the available resources and supports: “*New tools for revision were developed by [web-based educational resource] based on the Quebec school curriculum. These tools, targeted specifically to students, were the result of a collaboration between the teachers, the Quebec association of resource teachers, and the UNESCO chair for the curriculum development at [University name]*” (Translated from French) [43]. “*Those who need additional support to learn away from school or who need internet/technology access will be able to access supports at in-person and virtual study halls*” [44].

(1c) Safety and training of school staff to continue education provisions during the pandemic: Some policies described available supports and provided guidance for school staff working with children with disabilities so that their educational needs are fulfilled in a manner that is safe for both staff and children: “*resource teacher (IRT), caregivers and school administrators could have a need for additional protection equipment if they work with students with special needs (i.e., difficulties with liquids, drooling, sputum or excessive saliva, administering medication or other sanitary tasks)*” [32]. The supports offered to students and teachers included flexibility of school plans: “*School teams, Teaching Support Teams and Student Planning Teams as defined in the inclusive education policy will be in place to ensure that plans are flexible in terms of how programing and supports will be delivered to best support well-being and achievement*” [40].

#### 3.3.2. Article 11: Situations of Risk and Emergency

The second most common UN CRPD article was article 11, which relates to how individuals with disabilities must be considered in situations of emergency and elevated risk. The UN CRPD calls for the protection of persons with disabilities in situations of humanitarian risk [39], yet concerns have been raised over discrimination against persons with disabilities in Canada during the COVID-19 pandemic [45]. A total of 16 documents in our dataset were aligned with this article. There were six policies from MB and three or fewer from BC, SK, NS, NL, NB, NWT, and YK. Although the content was related to emergency response indicators in our categorization model, all of the extracts were in connection to education and school settings. Themes were: (2a) hygiene and preventative measures for institutions; (2b) funding and structural supports for institutions; and (2c) specific needs of children.

(2a) Hygiene and preventative measures for institutions: Documents outlined measures taken in relation to the pandemic, such as cleaning, physical distancing, and public transportation, that were specific responses to the emergency. For instance, MB policies emphasized protocols for increased cleaning of school spaces and increased busing capacity serving schools, and YK policy also had specific guidelines for physical distancing and cleaning in school bus services. 

A policy in MB provided details on exceptions to wearing masks in schools and considering safety for those who, for some reason, such as due to a disability, could not wear a mask: “*For those who are granted exceptions to mandatory mask wearing, it is important to continue practicing all the public health fundamentals, including staying home when ill, frequent hand washing with soap and water or alcohol-based hand sanitizer, covering coughs, and physical distancing*” [46].

In NL, guidance was provided for schools to set up a space for temporary isolation of staff or a student who becomes ill during the day.

(2b) Funding and structural supports for institutions: Examples of content related to this theme include a policy in MB describing access to special funding for schools to support the purchase of personal protective equipment for staff and students and increase service capacity for compliance with the public health emergency requirements: “*The funding will be used to support schools, teachers, and students across the province by providing masks and other personal protective equipment; enhancing cleaning and sanitization…*” [47].

(2c) Specific needs of children: Considerations for age that were specific for children, such as difficulty in maintaining social distancing and the need to maintain safe use of hand sanitizer amongst young children, were part of the recommendations in relation to the public health measures in SK, though not specifically for children with disabilities: “*For younger children, maintaining physical distance is less practical and the focus should be on minimizing physical contact instead. The precautionary measures within these guidelines will be implemented to reduce risk, and include standards for cleaning and sanitization along with measures for general operations, facilities, transportation and programming*” [48].

Other considerations for the specific needs of children were addressed in a policy from BC, outlining that some events, such as school dances and events, should be considered “essential services” because they were needed for child well-being and mental health, and therefore should be continued, following the public health guidance [49]. Some policies also addressed planning for the return-to-school after lockdown, including a policy from MB: “*School division and school plans include considerations for students with special needs and students at risk, consistent with inclusion and appropriate educational programming…*” [50].

#### 3.3.3. Article 19: Living Independently

Article 19 states that people with disabilities have the right to live in the community, and have opportunities to choose and enjoy communal spaces, including places of residence, and to receive in-home and community support services that prevent exclusion and segregation. These services should be offered to persons with disabilities according to their needs [39]. Policy documents that were aligned with Article 19 addressed areas related to (3a) Community service provisions and (3b) Recommendations for educational settings.

(3a) Community service provisions: Different resources available in the community that were impacted during the pandemic were captured in the Article 19 category. Community-based resources, such as respite care, support services for obtaining food and supplies, and mobility options, were some of the areas addressed in the policies. Financial supports for maintaining autonomy in the communities where people live were also identified. For example, a policy in MB addressed respite care: “*Respite remains an important short break from the unique demands of caring for a child with disabilities. It is available for caregivers of children who are eligible for supports from Children’s disability Services (CDS). For children who have lifelong complex medical needs, respite can be provided by a registered nurse or licensed practical nurse through the local regional health authority. Respite is not intended to replace academic learning. Respite can be provided in or out of the child’s home*” [50].

Disability services were mentioned in a document from AB, with recognition for the challenges in re-establishing services that were impacted during the pandemic: “*With Alberta’s relaunch strategy, the community disability service sector is preparing for a staged relaunch of disability services and supports that were reduced, provided virtually and/or closed to reduce the spread of COVID-19*” [51].

Direct community living supports during the pandemic, although not specific for children with disabilities, would also support health and well-being, such as food and medicine delivery: “*If you are a low-income senior or a person with a disability you can get: paid or subsidized delivery of meals, delivery of medicine and other necessities*” [52]. The same document specified that there were services provided for children with special needs available in the community, which should be discussed with the service providers.

Considerations for mobility and travel exemptions that could impact families of children with disabilities included: ”person supporting a PEI resident experiencing a severe injury, illness or disability permanent relocation due to severe illness or injury to be closer to a care provider attend and support a person in palliative care” [53].

Finally, financial resources offered to families could facilitate access to in-home and community-based services and were also captured under Article 19, as exemplified in this policy from ON: “*If you are a parent caring for a child with a severe disability, you may be able to receive financial support through the Assistance for Children with Severe disabilities Program. This program provides financial support for low- to moderate-income families to cover some of the extra costs of caring for a child who has a severe disability*” [54].

(3b) Recommendations for educational settings: From the amount of policy documents that matched Article 19 that were related to school settings, we can see that schools are often considered the main community setting for children. Some of the safety precautions in relation to children with disabilities in schools reflected aspects also captured in Article 24, such as the need for educators and school staff to adapt to the needs of children with disabilities while also responding to the public health measures: “*Supporting students with disabilities and diverse abilities may require those providing services to be in close physical proximity or in physical contact with a student for an extended period of time. Those providing these services should wear a non-medical mask when providing services when the service cannot be provided from behind a barrier*” [49].

Other documents captured under Article 19 outlined considerations for the needs of children with disabilities in the community, with particular emphasis on contamination risk in the initial stages of the pandemic and towards solutions to address these risks as services returned: “*As we plan for the safe and successful return to school for students with special needs and students at risk, it is important that students and families work with their student support team within the student-specific planning process*” [50]. In this context, we identified that community-based health services for children with disabilities are often offered in school settings and therefore were captured under Article 19: “*It [the policy] covers the six following domains: management, communications, material and information resources, educational services, supports to the students with disabilities or in challenges of adaptation and learning, and those with special needs, as well as mental health and well-being supports*” [43]. Additionally, community services may also be understood as types of supports offered in the school, as exemplified in this document from the YK: “*Resource programs for students with diverse learning needs and disabilities, transitions programs and other programs for students needing different supports will continue at school all day every day*” [44].

#### 3.3.4. Article 26: Habilitation and Rehabilitation

Article 26 emphasizes the services and programs needed “to enable persons with disabilities to attain and maintain maximum independence, full physical, mental, social and vocational ability, and full inclusion and participation in all aspects of life” [39]. The Article includes rehabilitation services in the areas of health, employment, education, and social services. Similar to what was captured in Article 19, the policies matching with Article 26 also highlight the need to build capacity among professionals and staff who provide these services and the provision of equipment and technology required to support rehabilitation. The related themes that were identified in the policy documents addressed: (4a) Rehabilitation programs offered in schools and the (4b) Establishment of alternative rehabilitation services.

(4a) Rehabilitation programs offered in schools: Our model captured aspects related to rehabilitation offered in school settings that encompass most of the indicators of structures and processes described in Article 26. Considerations were identified for individual schools and school districts to create the necessary structures and adapt educational and rehabilitation-related programming to respond to the needs of children with disabilities, as detailed in an MB document: “*Student-specific plans (e.g., adaptation plans, modification plans, individual education plans, behaviour intervention plans, health care plans, personal transportation plans) are key in supporting students with special needs and students at risk as they transition back to in-class learning. Student-specific plans may need to be reviewed and adjusted more frequently to ensure effective supports, strategies, and services are maintained or adjusted as the school year gets underway*” [50]. Similarly, in NL, a policy directed school districts to “*consider and enable the full participation and inclusion of students with exceptionalities. In circumstances in which supports and services require support and adaptation for public health measures, plans must be developed to ensure their inclusion*” [42].

Similar considerations for planning were also captured in policy documents from the Yukon: “It is recognized that educators’ instructional plans will need to be adaptable to meet any changing public health requirements, and that in the following circumstances not all students may be able to receive full-time in-class instruction at a school […] Behavior support plans, the provision of learning supports through adaptations must continue to be offered to the greatest extent possible” [33].

(4b) Establishment of alternative rehabilitation services: The offer of rehabilitation services is the focus of Article 26. During the pandemic, it was necessary to consider how these services could be continued, which included adaptation to remote services and changes in the services’ structures. Some policies highlighted the urgency of maintaining rehabilitation programs to avoid other health risks for persons with disabilities. Specific guidelines for autistic individuals and persons with physical and intellectual disabilities were created in QC. These guidelines included concerns for avoiding disease transmission, and preventing deconditioning and promoting a safe return to rehabilitation services, according to the time of the pandemic. These directives included specific recommendations, such as creating, expanding, and maintaining telerehabilitation options, optimizing home services, hiring university students to help with service provision, and offering the option for service providers to apply for funds to expand necessary supports [41]. Quebec and Manitoba had specific guidelines for rehabilitation services that included specific mention of mental health services [55]. Examples referred to re-structuring group programs in the rehabilitation setting to individual, home-based options and connecting to existing mental health services and supports: “*The mental health and well-being of Manitobans continues to be a shared priority of Manitoba Education and the Department of Families. Social-emotional learning, including self/co-regulation, response to trauma, and support for dealing with anxiety, will continue to be addressed in the classroom and/or through student-specific planning, as appropriate*” [48].

## 4. Discussion

Our analysis included 148 policy documents from 10 Canadian provinces and 3 territories. The policy documents included public health recommendations and regulations, informational support for different types of services and resources supported by provincial/territorial governments, and some described general recommendations or considerations for citizens and services during the COVID-19 pandemic. The UN CRPD model analysis showed that those policy documents were most frequently matched with Article 24 (Education); Article 11 (Situations of risk and humanitarian emergencies); Article 19 (Living independently and being included in the community); and Article 26 (Habilitation and Rehabilitation). Across the Articles, the predominant themes identified related to the provision of services in schools and the education sector.

Our mental health impact model captured a very limited number of policies addressing the mental health impact of the pandemic on children and youth. Some policies recognized the mental health risks that could be caused by disruptions in the routine of daily life and the long-term isolation during the pandemic. Of these policies, an even smaller number considered the specific needs of children and youth with disabilities and their families, and none of the policies identified proposed an action plan with specific services, structures, and mitigation strategies to alleviate these impacts or promote mental health during or after the pandemic.

Children, in general, were at higher risk for mental health conditions and multiple vulnerabilities and human rights violations before the pandemic [56]. For children with disabilities, the pandemic exacerbated existing gaps in services and supports and created new mental health constraints [57]. Restrictions to community living opportunities, including leisure and play, and limited access to school. In Canada, most health and social services and supports for children with disabilities are offered through the school settings [58,59,60]. The complete lack of access to these services during the lockdown and the chaotic return of these services accentuated the stress that families had to face and that, for an extended period of time, beyond the time that children without disabilities had to endure [61]. These challenges were reported across the globe in numerous commentaries and in cross-sectional study reports during the pandemic [62,63]. The experiences of negative emotions, uncertainty about the present (e.g., public health announcements and how they applied for children who, for example, could not wear masks, had multiple chronic conditions, or required individual medical attention for daily activities) and future (e.g., time to return to school and format for rehabilitation services); altered routines, including sleeping, eating, and physical activity cycles; and the overall state of stress and alarm that was lived globally, contributed to mental health challenges for children and adults [64].

The WHO report on Developmental Disabilities and Delay that was being collected in Canada when the pandemic was declared revealed that the majority of families of children with disabilities felt they did not have access to the necessary services to support their child’s physical and mental health [64]. Data from the national statistics agency (Statistics Canada) also identified that families of children with disabilities were more concerned with the well-being of their children than the parents of children without disabilities [60]. Parents also reported changes in their own work and daily routine schedules that prevented them from devoting full attention to their child’s online education. Consistently, caregivers reported that they did not receive adequate supports, such as respite care and health care supports for other chronic health conditions of their own or their child’s, which added to an overall poor mental health state at the family and for the children [21,65,66]. These considerations were acknowledged in the policies we identified, but in the absence of concrete action plans described in the policies, it is challenging to draw direct links between policy intentions and actual outcomes for the population.

Our group conducted a study with caregivers and youth with disabilities parallel to the collection of policies reported in the current manuscript [57]. In these reports, we saw that caregivers and youth with disabilities reported feeling stressed, lost, and deprived of basic services that were essential for their mental health. The lack of services reported by families encounters the intentions outlined in the policy documents, such as considerations for children with disabilities in schools, exceptions and considerations for mask wearing and adherence to public health protocols, and the priority return of services. Nevertheless, the statements identified in the documents do not clearly outline the application of these policies, and we cannot ascertain, from the policy documents alone, if the implementation responded to the immediate needs of families that were voiced in the different studies as needs not met. 

The UN CRPD and human rights treaties should serve as an instrument through which countries develop their policies and legislation at all times, including prior to and during public health emergencies. A rights-based approach to help can encompass the development of policies and the delivery of health services that address the social determinants of health and consider the upstream factors leading to better health and preventive measures that could prepare the systems of care in the case of a public health emergency [67]. The UN CRPD connection with the provision of health and social services is extended through General comment created by the UN CRPD committee, outlining, for example, how mental health policies should consider the UN CRPD provisions in considering persons with disabilities as having equal capacity before the law [68], suggesting that states should implement principles of the convention into their mental health laws. The use of the convention, as suggested, could support the promotion of health and rights for all at all times and should be a potent instrument in protecting the health of the most vulnerable populations, such as children with disabilities, during a crisis. It seems, however, that often the application of the convention occurs in a punitive approach: where the provisions are only considered in the absence of a service or when rights are violated [69], rather than services and laws being created based on a rights framework [70,71]. A gap exists between the policies, their application, and the perception of their application by families on the ground. 

In contrasting the policies identified in this study with the perspectives of families in the different surveys mentioned above, we can see a limited use of rights-based framework in policies [21,57,65,66]. For instance, families indicated that their children did not receive adequate supports for distance learning, policies recognized that “not all students will have the same type of access to essential educational services”, and yet the same policies did not propose a mitigation plan to address this inequitable service offer. The divide between policy intention and action is well known and has been thoroughly investigated [72,73]. Research-based evidence produced through interviewing the people being affected by policies and contrasting those lived experiences with actual policy propositions may be a path to connecting policy intentions to actions, informing policy coalitions, and shedding light on policy directions and priorities.

Compliance with rights-based approaches and the UN and WHO considerations [18,21,22], as stated in the Sendai Framework [74] and other guidance documents, can serve as a better structure for future policy planning and lead to less negative consequences in the aftermath of a pandemic. For instance, the intersectoral collaborations that are fairly established between health, education, and social services must be strengthened to guarantee equitable access to essential services normally provided through schools (such as food, social supports, and rehabilitation) when the school services are no longer available.

Learning from the pandemic, the high reliance on the school system to deliver a range of essential services and supports for children and families must be questioned. This tendency is illustrated by the policies identified in this study and the dominance of educational policies in the dataset. Suggestions for restructuring health services for children with disabilities are not new [75]. While the school system is often the main safety net where children are accounted for, we must consider where and how the health and community systems should also assume responsibility: increasing capacity in the community to care for children with complex needs, creating better structure and services for respite care for families, including strengthening of formal and informal networks and structures of support and distributing financial supports across sectors—from health and education to community may be essential steps in restructuring services as we recover from the pandemic. Building a stronger community-based structure for services and supports would be a structure that would favour mental health outcomes and general positive outcomes for children and families [76].

Other structural barriers that we can identify from the analysis of the policies in this study point to the need to build the capacity of the population in general and healthcare workers in relation to the needs of children with disabilities and their families. Most policy recommendations were addressed to educational settings/staff, but few guidelines addressed healthcare workers on the frontlines in relation to testing and providing information for autistic children or children with intellectual disabilities and their families, for example, a population that should receive consideration in all types of services. The need to collect continuous data on children with disabilities in Canada has been highlighted by the UN Concluding Observations to Canada [77]. Systemic, disaggregated data collection on children with disabilities and their families could allow for strategic investments in the training of professionals across sectors, efficient service delivery, and planning for access to services, such as the internet, home-based resources, such as computers and assistive technology devices, and families capacity to support their child health and learning [26].

Little was mentioned in the policy documents about the engagement of children and youth and their families in the development of policies and recommendations. The engagement of persons with disabilities, including children, and their representative organizations through Non-Governmental Organizations, OPDs, and CSOs is a priority in the recommendations for the implementation of the UN CRPD [78] and patient engagement in research and service delivery is promising in generating better health services and systems [79]. Another study completed by our group identified a few countries that had consulted persons with disabilities to advise on pandemic-related policy responses [30]. In Canada, the Disability Advisory established at the beginning of the pandemic supported guidance for disability-inclusive responses. However, the systems where children and families transit and use (i.e., schools and leisure facilities) were often not included in these recommendations [80]. The UNICEF report on children with disabilities during the pandemic highlighted that most countries had not engaged disability persons organizations in disability-inclusive responses. During the pandemic, Canada was in the process of reporting to the UN Committee on the Rights of Children. An additional parallel report specifically on the COVID-19 scenario for children with disabilities warned of the increased gaps in health, education, mental health, and family supports created during the pandemic [20,27,30,81] and brought to attention the need to engage youth-based groups, families and bring together disability and children’s rights organizations in planning and advising for a policy that is inclusive of the needs of children with disabilities. Another report on the “10 top threats for Canadian Children” puts mental health and youth engagement as some of the priorities to consider in informing policy and programs [82]. As a result, it is crucial to ensure that this recommendation is respected even in the implementation of policies targeted at other priorities. Engaging youth and their families is the most viable strategy for addressing the gap identified above between existing policies and reported family needs. Creating a strong consultation structure that includes youth with disabilities and their families and professionals involved in inter-sectoral services could be a pathway to creating and implementing inclusive and more effective policies and services.

Lastly, we should consider that in the Canadian federalist system, most policies that affect children with disabilities and their families are of provincial jurisdiction, which is the reason why we analyzed the policies at that level. From the UN CRPD implementation perspective, it is important to understand that provinces have the obligation to apply the principles of the convention to their policies. It is expected that where provincial disability acts are in place, the policies for persons with disabilities would be better in areas, such as accessibility of the built environment (e.g., for testing and vaccine facilities). However, our analysis was not conclusive on that front. Future exploration about the institutions and governance structures that can have an impact on policymaking for children may be necessary to suggest areas where awareness raising, capacity building, and collaborations may be needed between government sectors, civil society, and academia to strengthen a systemic application of human rights into all policies.

### Limitations and Future Directions

This study included policies collected in relation to the COVID-19 pandemic during an 8-month period. A longitudinal data collection may have generated a more comprehensive representation of how policy responses evolved across the different pandemic waves. However, we believe the policies collected between the first lockdowns and the first “return” to activities may offer a good representation of governments’ preparedness to create inclusive and equitable emergency responses and shed light on the use or lack of rights-based approaches in these responses. A systematic approach to human rights monitoring, as suggested by the UN OHCHR, bringing together researchers, persons with disabilities, including children and families, and governments would facilitate an ongoing, longitudinal approach to addressing human rights in public policy [77].

We also recognize that although text mining methodology is effective and frequently used to analyze large bodies of text, it is rarely used with a complex dictionary and categorization model, as we did. We used this innovative approach to text mining in order to capture if and in what instances the policy document reflected the language of the UN CRPD and followed this first screening with a manual thematic analysis. We recognize that we might have missed content using the screening process; however, through our multiple layers of validation and manual coding, we are confident that these results can reflect the overall approach to mental health and the key Articles of the UN CRPD. This approach can be of relevance for monitoring the presence of rights-based language and indicators in policy development, including in comparison to the policy documents produced during the COVID-19 pandemic.

## 5. Conclusions

Children and youth with disabilities and their families and caregivers have experienced important impacts of the pandemic in their daily lives and on their mental health. Some of these impacts have been acknowledged in policy documents produced by different sectors of provincial and territorial governments in Canada, but few action plans were identified to mitigate the impact. Education sector policies reported most of the possible impacts for children and are aligned with aspects related to Emergency Responses, Education, Community Living, and Rehabilitation Services Articles of the UN CRPD. Considering a cross-sectoral approach that enables communities to respond effectively to the mental health promotion of children with disabilities and their families and adopting a broader understanding of human-rights-based approaches to health will be important steps in future emergency planning and current policy development.

## Figures and Tables

**Table 1:** Data extracted from policy documents.

Category	Details
Inclusion criteria:	If the policy follows all the inclusion criteria, please add YES, go to the next column, “Excluded”, add NO, and continue your data abstraction. If the policy does not follow the inclusion criteria, please add NO in the “Inclusion criteria” column and use the next column, “Excluded”, to add the rationale for exclusion (e.g., published in 2018 before the pandemic). Then, stop abstracting data from this policy as it would be excluded, and start with the next policy.
Policy type	Please use the dropdown menu to classify the policy. If the policy does not fit under the classification provided, select “other”.
Policy lever	Policy levers are mechanisms available to decision-makers to influence system changes. Please select from the dropdown menu (e.g., legislative, administrative, regulatory, and other).
Government level	Please add what level of government or institution will implement the policy (e.g., local, municipal, provincial and federal government).
Operational details	How does the policy work/operate? Please select from the dropdown menu: YES, if it is mandatory; NO, if it is not mandatory; and NR, if it is not reported.
Funding	Add information on how the policy is funded. If not funded, please add NO. If not reported, please add NR.
Policy goals	Please copy and paste the goals/objectives of the policy.
Context	Please add in which context the policy was created (e.g., out-of-pocket expenses with healthcare increased due to COVID-19; therefore, this policy was created to ensure….; historical context, such as the policy has been debated previously because….). This information usually is given in the introduction section.
Implementation mechanism	Please use the dropdown menu to select your answer (e.g., YES or NO)
Implementation mechanism details	If you selected YES in Q9, please copy and paste the implementation mechanism as reported by the authors (e.g., online counselling will be funded by the health ministry; and respite care workers will have priority in immunization to continue services supports for families). If you selected NO in Q9, add “NO” (please do not leave blank cells).
Short-term outcomes	Please add the expected short-term outcomes.
Intermediate-term outcomes	Please add the expected intermediate-term outcomes.
Long-term outcomes	Please add the expected long-term outcomes.
Costs	Please add any costs related to the policy. If not reported, add NO.

**Table 2:** Documents identified per province.

Location (*n* = 13)	Polices Included (*n* = 148)
British Columbia (BC)	12
Alberta (AB)	10
Saskatchewan (SK)	3
Manitoba (MB)	26
Ontario (ON)	22
Quebec (QC)	38
New Brunswick (NB)	6
Newfoundland and Labrador (NL)	10
Nova Scotia (NS)	5
Prince Edward Island (PEI)	7
New Territories (NT)	3
Nunavut (NV)	1
Yukon (YK)	5

**Table 3:** UN CRPD articles are captured in the policy documents.

Article	Themes Identified in Thematic Analysis (Only for Articles with Greater than 25 Frequency)	Provinces
Article 24: Education	Services provided in a school setting and intersectionality Considerations for alternative learning methods safety and training of school staff to continue education provisions during the pandemic	AB, BC, NL, NS, QC, SK, NU, YT
Article 11: Humanitarian Emergencies	Hygiene and preventative measures for institutions Funding and structural supports for institutions Specific needs of children.	BC, NB, NL, NS, SK, NU, NWT, YT
Article 19: Independent Living	Community service provisions Recommendations for educational settings	AB, BC, MB, NL, ON, PEI, QC, YT
Article 26: Habilitation and Rehabilitation	Rehabilitation programs offered in schools Establishment of alternative rehabilitation services	MB, NL, QC, YT

## Data Availability

The data sources used and/or analyzed during the current study are available in the Appendix, and full policy texts are available from the corresponding author upon reasonable request.

## Appendix A

List of government websites searched during data collection:

ALBERTA

Ministry of Community and Social Services

Ministry of Children’s Services

Ministry of Community and Social Services

Alberta Emergency Management Agency

BRITISH COLUMBIA

Ministry of Social Development and Poverty Reduction

Ministry of Children and Family Development

Ministry of Public Safety and Solicitor General

Emergency Management BC

MANITOBA

Department of Families

Department of Families

Department of infrastructure

NUNAVUT

Department of Education

Department of Family Services

Department of Family Services

Department of community and government services

Nunavut Emergency Management

ONTARIO

Ministry for Seniors and Disability

Ministry of Children, Community, and Social Services

Ministry of the Solicitor General

SASKATCHEWAN

Ministry of Social Services

Office of Disability Issues

Ministry of Social Services

Ministry of health

Saskatchewan Public Safety Agency

YUKON

Department of Health and Social Services

Department of Health and Social Services

Department of Community Services

## Appendix B

Screening form for documents to be retained for analysis:
  1. Policy Name
  2. Link
  3. Q1. Does this policy pertain to persons with disabilities?
  4. Q2. Is this policy inclusive of persons with disabilities?
  5. Decision (Include/Exclude)
  6. Reason for exclusion
  7. Comments

## Appendix C

Visualization of Mental Health Analytical Model
  - Mental health
    ◦ Stressors
    ◦ Barriers
      ▪ Environmental barriers
      ▪ Structural barriers
      ▪ Attitudinal barriers
      ▪ Medical barriers
    ◦ Symptoms and outcomes

## Appendix D

Visualization of UN CRPD Analytical Model (Examples using selected articles)
  - UN CRPD
    ◦ Article 6: Women and Girls
      ▪ Structure
        - Nondiscrimination and equality
        - Full development, advancement, and empowerment of women
      ▪ Process
        - Nondiscrimination and equality
        - Full development, advancement, and empowerment of women
      ▪ Outcomes
        - Nondiscrimination and equality
        - Full development, advancement, and empowerment of women
    ◦ Article 7: Children with Disabilities
      ▪ Structure
        - Equality and identity
        - Best interest for the child and respect for evolving capacities
        - Respect for the views of the child
      ▪ Process
        - Equality and nondiscrimination
        - Survival, development, and preservation of identity
        - Best interests of and respect for the views of the child
      ▪ Outcomes
        - Equality, identity, and best interest of the child
        - Respect for the views of the child
        - Article 11: Situations of Risk and Humanitarian Emergencies
      ▪ Structure
        - Prevention and response
        - Recovery, reconstruction, and reconciliation
      ▪ Process
        - Prevention and preparedness
        - Rescue and response
        - Recovery, reconstruction, and reconciliation
      ▪ Outcomes
        - Prevention and preparedness
        - Rescue and response
        - Recovery, reconstruction, and reconciliation
    ◦ Article 23: Respect for Family
      ▪ Structure
        - Nondiscrimination in family life
        - Parental rights of persons with disabilities
        - Right of children with disabilities to grow up in a family environment within the community
      ▪ Process
        - Family life and parental rights
        - Right of children with disabilities to grow up in a family environment within the community
      ▪ Outcomes
        - Nondiscrimination in family life
        - Parental rights of persons with disabilities
        - Right of children with disabilities to grow up in a family environment within the community
    ◦ Article 24: Education
      ▪ Structure
        - Inclusive education
        - Quality and free primary and secondary education
        - Access to tertiary education, vocational training, and lifelong learning
        - Inclusive teaching
      ▪ Process
        - Inclusive education
        - Quality and free primary and secondary education
        - Access to tertiary education, vocational training, and lifelong learning
        - Inclusive teaching
      ▪ Outcomes
        - Inclusive education
        - Quality and free primary and secondary education
        - Access to tertiary education, vocational training, and lifelong learning
        - Inclusive teaching
